# Improvement of gut microbiome and intestinal permeability following splenectomy plus pericardial devascularization in hepatitis B virus-related cirrhotic portal hypertension

**DOI:** 10.3389/fimmu.2022.941830

**Published:** 2022-09-08

**Authors:** Yang Zhao, Rui Zhou, Ying Guo, Xi Chen, Aiyu Zhang, Jiayin Wang, Fanpu Ji, Bowen Qin, Jing Geng, Guangyao Kong, Zongfang Li

**Affiliations:** ^1^ Shaanxi Provincial Clinical Research Center for Hepatic & Splenic Diseases, The Second Affiliated Hospital of Xi’an Jiaotong University, Xi’an, China; ^2^ National & Local Joint Engineering Research Center of Biodiagnosis and Biotherapy, The Second Affiliated Hospital of Xi’an Jiaotong University, Xi’an, China; ^3^ Department of Hepatobiliary Surgery, The Second Affiliated Hospital of Xi’an Jiaotong University, Xi’an, China; ^4^ School of Computer Science and Technology, Xi’an Jiaotong University, Xi’an, China; ^5^ Department of Infectious Diseases, The Second Affiliated Hospital of Xi’an Jiaotong University, Xi’an, China

**Keywords:** liver cirrhosis, portal hypertension, splenectomy, gut microbiome, intestinal permeability

## Abstract

The gut microbiome is an essential component of the intestinal mucosal barrier, critical in regulating intestinal permeability. Microbiome dysbiosis and intestinal permeability changes are commonly encountered conditions in patients with cirrhosis and are closely related to its development and further complications. However, alterations in the gut microbiome and intestinal permeability in chronic hepatitis B virus (HBV) patients with cirrhotic portal hypertension after undergoing a splenectomy plus pericardial devascularization (SPD) have not been investigated. This study recruited 22 patients who were measured against themselves on the study parameters before and after an SPD, along with 20 healthy controls. Methodologically, fecal samples were collected for gut microbiome analysis by 16S ribosomal DNA sequencing, and peripheral blood samples were obtained to examine the liver function and intestinal permeability. This study showed that the community structure of the gut microbiomes in patients before the SPD exhibited obvious differences from those in the healthy control group. They also exhibited a decreased bacterial community richness, increased intestinal permeability, and enhanced inflammation compared with the healthy controls. These issues were further aggravated two weeks after the SPD. There was also evidence of significantly higher abundances of *Streptococcaceae*, *Enterobacteriaceae*, and *Enterococcaceae* than those in the healthy control group. However, 12 months after the surgery, 12 of the 16 patient-associated genera recovered, of which 10 reached normal levels. Additionally, the microbiome diversity increased; the bacterial composition was back to a level similar to the healthy controls. Liver function, intestinal permeability, and inflammation levels all improved compared with preoperative levels. Furthermore, correlation analyses indicated that the five recovered bacterial taxa and the Shannon diversity index were correlated with several improved clinical indicators. Altogether, the improvements in the liver function and intestinal permeability in HBV-related cirrhotic patients may be related to the restoration of the gut microbiome after an SPD.

## Introduction

As the pathologic end-stage of advanced liver disease from hepatitis B virus (HBV) infection (1), cirrhosis may unavoidably progress to portal hypertension, which subsequently leads to various portal hypertension-caused complications, such as hypersplenism, gastroesophageal varices, variceal hemorrhage, and ascites (2). Hypersplenism is the most common complication, with an incidence rate of approximately 64% (3). Its presence indicates a more advanced stage of liver disease and an increased risk of complications. A splenectomy plus pericardial devascularization (SPD) is a classic and efficacious surgical therapy to alleviate pancytopenia caused by hypersplenism, improve liver function, and reduce portal pressure and the risk of variceal hemorrhage (4, 5). However, many postoperative complications, such as infection and thrombosis, are the most common threats to post-SPD patients (6). Therefore, analyzing the relevant risk factors for postoperative complications and strengthening perioperative management are crucial to improving a prognosis.

The gut microbiome refers to a wide variety of microorganisms, predominantly bacteria, that reside in the host’s gastrointestinal tract. The gut microbiome can maintain normal intestinal barrier function by protecting the intestines from colonizing and invading pathogens and producing beneficial metabolites (7). The liver is the first extraintestinal organ to receive venous blood from the gut *via* the portal vein. It communicates bidirectionally with the gut and its microbiome through the gut-liver axis (8). Liver dysfunction in patients with cirrhosis can negatively affect the gut by reducing bile acid secretion, impairing intestinal motility, incurring portal hypertension, or decreasing the synthesis of antibacterial molecules (9, 10), all of which may cause a changed intestinal microenvironment, further leading to dysbiosis of the gut microbiome and alteration in intestinal permeability (11). The gut microbiome dysbiosis begins before cirrhosis development and during the progression of chronic liver disease. The severity of the disorder has been found to correlate with the degree of liver function damage present at the time (12). The alteration of the gut microbiome in patients with cirrhosis is usually characterized by an overgrowth of potentially pathogenic bacteria concomitant with a decrease in the levels of beneficial bacteria (13, 14). Gut microbiome dysbiosis and intestinal barrier injury significantly contribute to the progression of cirrhosis and have also been implicated in the pathogenesis of cirrhosis-related complications (15). Previous studies have confirmed the relationship between the perioperative or postoperative gut microbiome characteristics and prognoses (16, 17). Certain specific bacterial taxa have been identified as independent risk factors for the adverse clinical outcomes of patients (18, 19). All this evidence suggests that maintaining the dynamic balance of a normal gut microbiome may represent a promising approach to alleviating postoperative complications and improving the prognosis after an SPD. However, the gut microbiome can vary with different etiologies of liver cirrhosis. The alterations in the gut microbiome and intestinal permeability in HBV-related cirrhotic patients after undergoing an SPD are yet to be reported.

In the study, we evaluate the gut microbiome and intestinal permeability status between healthy controls and HBV-related cirrhotic patients with portal hypertension and hypersplenism. In particular, the differences in intestinal microbial communities before and after the SPD were characterized. Correlations between specific bacterial taxa as well as liver function and intestinal permeability in the patients were also analyzed. The present study could help to gain a better understand of the risks and beneficial effects of SPD for cirrhotic patients from the perspective of their intestinal microenvironments.

## Materials and methods

### Inclusion and exclusion criteria

The patients in the study had HBV-related cirrhosis with portal hypertension and hypersplenism and had undergone an SPD procedure at the Second Affiliated Hospital of Xi’an Jiaotong University. The inclusion criteria were designated as follows: [1] The patients had been diagnosed according to the guideline of prevention and treatment for chronic hepatitis B in China (2015 update) by comprehensive consideration of liver biopsy results, imaging examinations, clinical features, physical signs, laboratory tests, medical histories, progress notes, and associated complications (20). [2] All the patients suffered varying degrees of splenomegaly, and the majority of them had moderate or severe esophagogastric varices as revealed by upper gastrointestinal radiography or endoscopy examinations. [3] The clinical indications for an SPD included endoscopic treatment-resistant esophagogastric varices with or without variceal hemorrhage, history of esophageal variceal bleeding or potential bleeding or infection due to hypersplenism and thrombocytopenia (platelet count <50×10^9^/L), and upper abdominal discomfort owing to an enlarged spleen (5, 6). [4] The patients were not treated at the hospital until their stool and serum samples had been obtained.

The exclusion criteria for this study were detailed as follows: [1] Patients who presented with hepatic carcinoma, hepatic encephalopathy, or preoperative Child-Pugh class C were excluded. [2] Patients who concomitantly suffered from other disease entities (such as diabetes, hypertension, obesity, metabolic syndrome, inflammatory bowel disease, nonalcoholic fatty liver disease, coeliac disease and cancer) were excluded. [3] Patients who had received antibiotics and/or probiotics within the three months of the onset of the study were also excluded.

The inclusion and exclusion criteria of the healthy control group were set as follows: [1] The healthy individuals underwent routine health checkups in the Second Affiliated Hospital of Xi’an Jiaotong University and did not fulfill the exclusion criteria listed above. [2] The results of liver imaging, liver biochemistry, physical examinations, urine, blood, and stool tests were within the normal range. [3] Participants in this group were selected by matching them with the study patients based on their age, sex, and body mass index score.

All patients were informed about the benefits and risks of SPD, and prior informed consent was obtained from all participants. The study conformed to the ethical guidelines of the 1975 Declaration of Helsinki and was approved by the Institutional Ethics Committee of the Second Affiliated Hospital of Xi’an Jiaotong University (Approval No. 2017-416).

### Fecal sample collection, DNA extraction, and PCR amplification

Fresh fecal samples from patients with cirrhosis (before the SPD as well as two weeks and 12 months after the SPD) and healthy individuals were collected in a sterile container and delivered immediately from the hospital to the laboratory using an insulated polystyrene foam box filled with ice. Upon collection, each stool sample was immediately divided into aliquots, flash-frozen in liquid nitrogen, and stored at -80°C before analysis. Total bacterial DNA was then extracted from a frozen aliquot (200 mg) of each fecal sample using a QIAamp DNA Stool Mini Kit (51504, Qiagen, Germany) in accordance with the manufacturer’s instructions. The quality and quantity of the DNA were measured considering ratios of 260/280 nm and 260/230 nm using a NanoDrop spectrophotometer (NanoDrop 2000, Thermo Scientific, Wilmington, DE, USA). The V3+V4 hypervariable region of the bacterial 16S ribosomal RNA (rRNA) gene was amplified with the common primer pair 338 F (5′-ACTCCTACGGGAGGCAGCAG-3′) and 806 R (5′-GGACTACHVGGGTWTCTAAT-3′) combined with adapter and barcode sequences. The thermal cycling conditions were as follows: initial denaturation at 95°C for 5 min (1 cycle), followed by 95°C for 30 s, 50°C for 30 s, and 72°C for 40 s (25 cycles), and a final extension at 72°C for 7 min.

### DNA library construction and sequencing

Purified amplicons were quantified by a Quant-iT™ dsDNA High-Sensitivity Assay Kit (Q33120, Invitrogen, USA) and pooled in equimolar amounts. Then, DNA libraries were constructed in accordance with the manufacturer’s (Illumina) instructions and sequenced on an Illumina HiSeq 2500 platform (Illumina, San Diego, CA, USA) with the paired-end 250 mode (2×250 bps) following the standard protocols provided by Biomarker Technologies Co. Ltd. (Beijing, China).

### Microbiome analysis

After excluding the adaptor and primer sequences, the raw sequences were assembled for each sample according to the unique barcode using the Quantitative Insights Into Microbial Ecology platform (QIIME, V.1.8.0). The raw paired-end reads from the original DNA fragments were merged by FLASH (V.1.2.7), and assigned to each sample according to the unique barcodes. All the effective reads from each sample were assigned to the same operational taxonomic units (OTUs) based on a cut-off of 97% similarity according to the UCLUST algorithm. For alpha diversity analysis, the OTUs were rarified to different metrics to analyze species diversity in a sample. This included generating curves for OTU rank, rarefaction, and the Shannon index. The standard Shannon and Simpson diversity indices and richness indices (including the Chao1 and abundance-based coverage estimator [ACE] indices) were calculated by Mothur (V.1.30). For beta-diversity analysis, principal component analysis (PCA), principal coordinate analysis (PCoA) and nonmetric multidimensional scaling (NMDS) were performed using the QIIME to evaluate differences in species complexity among the samples. All analyses were carried out with a bioinformatic pipeline tool, BMK Cloud (http://www.biocloud.net/).

### Enzyme-linked immunosorbent assay

Peripheral venous blood from each individual was collected into pro-coagulation tubes before and after the SPD. The tubes were left undisturbed at room temperature for 30 min and then centrifuged at 2,000 rpm for 10 min at 4°C. The supernatants (serum) were divided into aliquots and stored at -80°C until subsequent analysis. One aliquot was used for each assay to avoid multiple freeze/thaw cycles. The serum concentrations of tumor necrosis factor α (TNF-α), diamine oxidase (DAO), lipopolysaccharide (LPS), and D-lactate (D-LA) were measured by enzyme-linked immunosorbent assay (ELISA) kits (MLbio, Shanghai, China) in accordance with the manufacturer’s protocols. All samples were tested in triplicate. The optical density at 450 nm was measured using a microplate reader (PowerWave XS2, BioTek, Winooski, VT, USA).

### Statistical analysis

All statistical analyses were performed with the SPSS 21.0 statistical package (SPSS, Chicago, IL, USA). Values are presented as the mean ± standard deviation for normally distributed data or median and interquartile range for continuous variables following non-normal distribution or number (%) for categorical variables. One-way ANOVA test was used for comparison of continuous data among multiple groups, while the LSD-t test was used for further comparison between two groups. The Kruskal-Wallis test was performed to process the data that retained a non-normal distribution even after log transformation. The inter-group difference was compared with Fisher’s exact test for categorical variables. Multiple hypothesis tests were adjusted using the Benjamini and Hochberg false discovery rate (FDR); significant differences were considered when the results were below an FDR threshold of 0.05. Spearman’s rank correlation coefficient (*P*-value<0.05) was used to evaluate the associations between bacterial abundance and clinical characteristics as appropriate. All tests for significance were two-sided, and *P*<0.05 was defined as statistically significant. All figures were plotted by Origin Pro8.0 software (OriginLab, Northampton, MA, USA) and R software (V. 3.4.4).

## Results

### Study population

**Figure 1:**
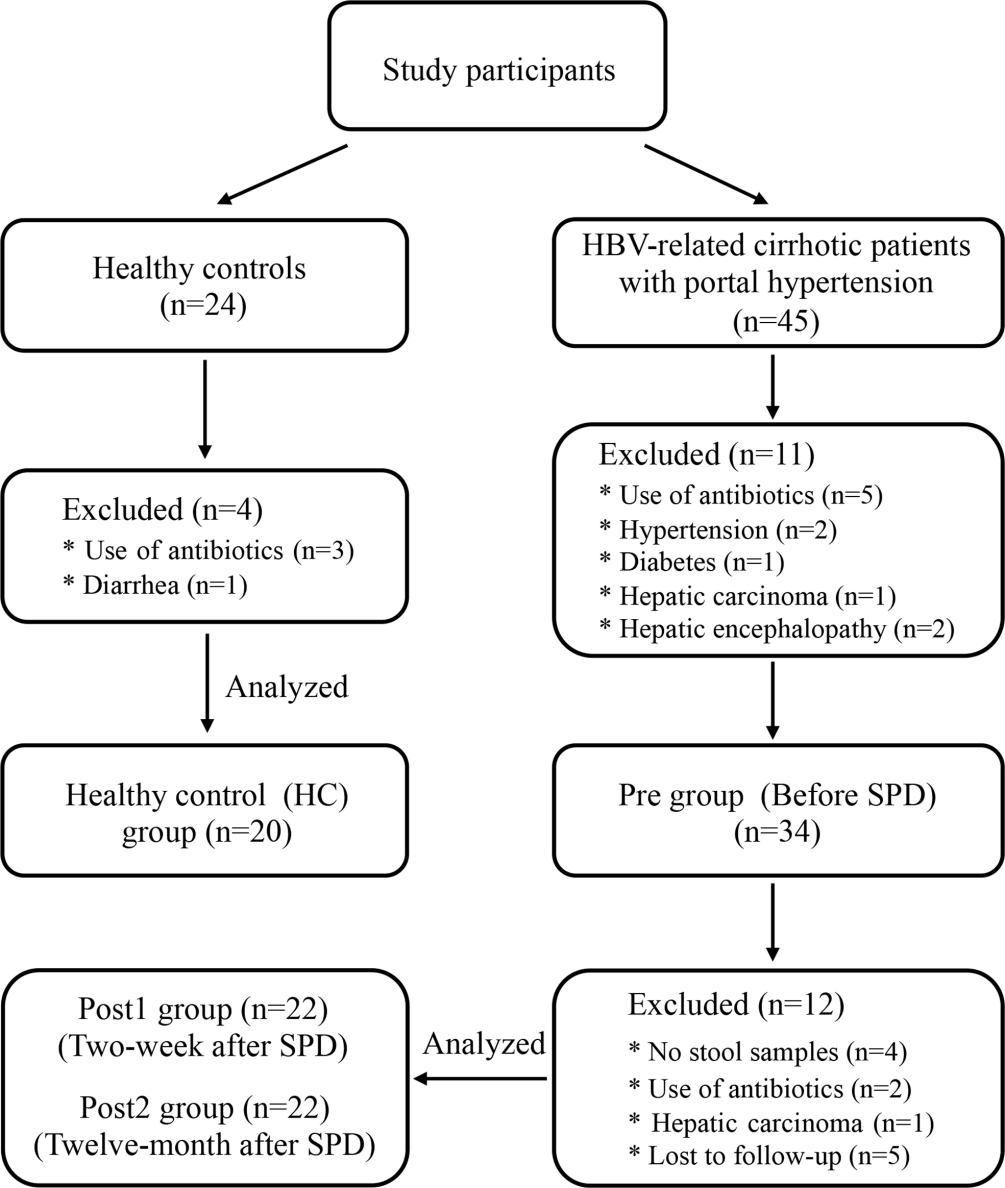
Flow chart of participants through each stage of the clinical study.

From March 1, 2017, to December 31, 2018, a total of 34 HBV-related cirrhotic patients who met the inclusion-exclusion criteria were enrolled in the study and were prepared for comparisons to themselves on the study parameters taken before the SPD (Pre) vs. two weeks after the SPD (Post1) vs. 12 months after the SPD (Post2) (**Figure 1**). Eight patients were excluded 12 months after the SPDs for the following reasons: One patient with cirrhosis had developed hepatocellular carcinoma, two patients had taken antibiotics within the three months prior to stool sample collection, and five patients were lost to follow-up. In summary, serum and fecal samples were obtained from 22 patients before and after the SPD (the stool samples of four patients were not collected at two weeks after the surgery) and from 20 healthy controls (HC). The clinical characteristics of the patients with cirrhosis and the healthy controls are shown in **Table 1**. As expected, the liver function of the cirrhotic patients with portal hypertension and hypersplenism was severely impaired, and blood cell counts were remarkably reduced compared with those of the healthy control group. However, the Child-Pugh classes of five patients at Post1 and eight patients at Post2 were downgraded from class B to A, with the decreases in the Child-Pugh scores from 6.2 ± 1.3 (Pre) to 5.8 ± 0.7 (Post1) (*P*>0.05) and 5.1 ± 0.4 (Post2) (*P*<0.001), respectively. Furthermore, blood cell counts increased after SPD and reached normal levels in the Post2 group. Therefore, liver function and pancytopenia were ameliorated significantly in the long term after therapeutic SPD.

**Table 1:** Clinical information summary of cirrhotic patients and healthy controls.

Characteristic	A group	B group	C group	D group	*P* value				
	HC (*n *= 20)	Pre (*n *= 22)	Post1 (*n *= 22)	Post2 (*n *= 22)	A vs B	A vs C	A vs D	B vs C	B vs D
Age	48.1 ± 9.1	46.2 ± 9.0	46.2 ± 9.0	47.2 ± 9.0	n.s.	—	—	—	—
Male/female	12/8	13/9	13/9	13/9	n.s.	—	—	—	—
BMI (kg/m^2^)	22.1 ± 1.3	22.0 ± 1.2	21.4 ± 1.1	22.0 ± 1.3	n.s.	n.s.	n.s.	n.s.	n.s.
TB (μM/L)	10.7 ± 3.0	25.7 ± 10.4	18.0 ± 9.1	16.7 ± 6.6	***	**	*	**	***
ALT (IU/L)	23.2 ± 8.9	36.5 ± 18.6	26.8 ± 17.6	31.1 ± 12.7	**	n.s.	n.s.	*	n.s.
AST (IU/L)	20.9 ± 5.0	41.1 ± 24.0	31.6 ± 14.6	38.5 ± 14.1	***	*	**	n.s.	n.s.
Albumin (g/L)	43.5 ± 4.4	36.3 ± 5.7	37.8 ± 5.7	42.0± 4.3	***	***	n.s.	n.s.	***
PT (s)	11.9 ± 1.1	13.3 ± 1.6	12.0 ± 1.1	12.1 ± 1.4	**	n.s.	n.s.	**	**
WBC (10^9^/L)	6.2 ± 1.2	2.2 ± 0.9	7.8 ± 2.2	5.9 ± 2.1	***	**	n.s.	***	***
PLT (10^9^/L)	227.9 ± 30.2	36.0 ± 13.3	303.3 ± 100.3	233.2 ± 69.9	***	***	n.s.	***	***
Ascites (*n*, %)	—	7 (31.8)	3 (13.6)	2 (9.1)	—	—	—	n.s.	n.s.
Child-Pugh score	—	6.2 ± 1.3	5.8 ± 0.7	5.1 ± 0.4	—	—	—	n.s.	***
Child-Pugh class (A/B/C)	—	14/8/0	19/3/0	22/0/0	—	—	—	**	**

BMI, body mass index; TB, Total bilirubin; ALT, alanine transaminase; AST, aspartate aminotransferase; PT, prothrombin time; WBC, white blood cell count; PLT, platelet count. *P < 0.05, **P < 0.01, ***P < 0.001; n.s. represents no significant difference. Data are expressed as mean ± standard deviation. Numbers in parenthesis are %.

### The changes in the microbiome diversity and bacterial composition 12 months after the SPD

**Figure 2:**
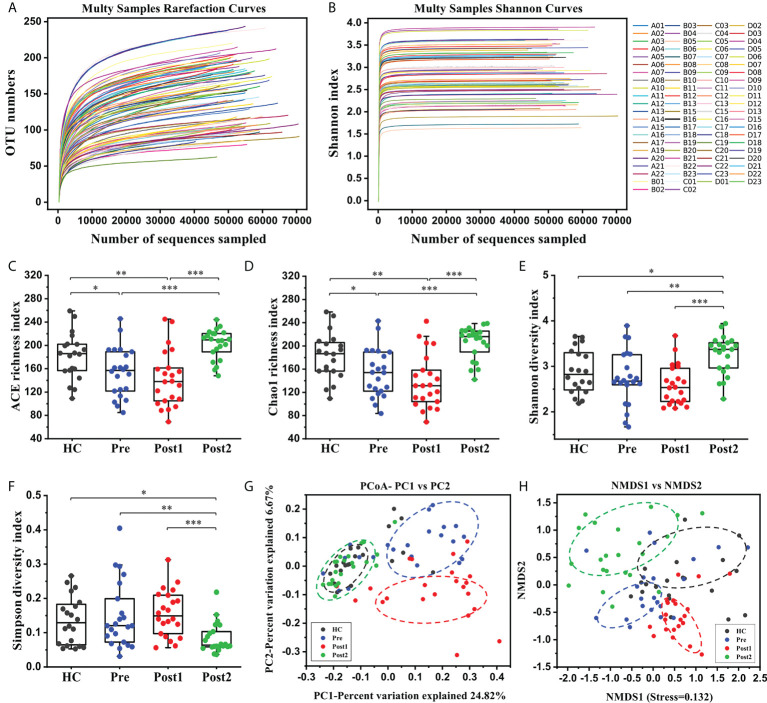
The alterations in the gut microbiome diversities and structures in patients after the SPD. **(A, B)** Rarefaction curves and Shannon curves of the gut microbiome in each sample. **(C–F)** Comparisons of the microbiome alpha diversity at Pre (*n *= 22), Post1 (*n *= 22), Post2 (*n *= 22), and in the healthy control (*n *= 20) groups. Alpha diversity was illustrated by the ACE richness index, Chao1 richness index, Shannon diversity index, and Simpson diversity index. The boxes represent the 25th through the 75th percentile, and the median value is shown as a horizontal line inside the box; the whiskers extend to 1.5 times the difference between the 25th and 75th percentiles. *P < 0.05, **P < 0.01, ***P < 0.001. **(G, H)** PCoA score plot based on Binary Jaccard and NMDS score plot based on Weighted Unifrac.

First, overall differences in the microbial community structures in the healthy controls and cirrhotic patients before and after SPD were calculated. High-throughput sequencing of the bacterial 16S rRNA gene V3+V4 regions in 86 samples produced 6,438,544 raw reads (an average of 74,866 reads per sample). After filtering the low-quality sequences and chimeras, 4,889,963 effective tags were obtained for the following analysis. Based on a 97% similarity level, all effective tags were clustered into OTUs. The rarefaction curve and Shannon index curve were plotted to reflect sequencing depths. As shown in **Figures 2A, B**, OTU numbers and Shannon indices reached plateaus with increases in sample sequence numbers, suggesting that the sequencing depth was adequate.

The alpha diversity of the gut microbiome was assessed using richness and diversity indices. The ACE and Chao1 indices, which measure species richness, revealed that the richness was significantly lower at Pre compared with that in the healthy control group (*P*<0.05), and richness was further reduced at Post1 (**Figures 2C, D**). However, richness was increased at Post2 compared with Pre and Post1 (*P*<0.001). The Shannon and Simpson indices, which reflect bacterial diversity, are influenced by both the richness and evenness of the community, and a relatively high Shannon index or relatively low Simpson index indicates an increased diversity. As shown in **Figure 2E**, the Shannon diversity index was slightly lower at Pre than in the healthy control group, but the difference was not statistically significant (*P*>0.05); however, this index was further decreased at Post1 than in the healthy control and Pre groups, and was restored at Post2 (*P*<0.05). Accordingly, the Simpson diversity index indicated the opposite tendencies during the whole process (**Figure 2F**). The above results indicated that a low richness and diversity of the gut microbiome existed in patients before undergoing the SPD and during hospitalization. However, this situation improved 12 months after surgery.

The beta diversity of the microbiome was assessed using unsupervised multivariate statistical methods, including PCoA (based on Binary Jaccard) and NMDS (based on Weighted Unifrac). The results showed that the bacterial compositions in the Pre and Post1 groups not only clearly deviated from each other but also set apart from those in the healthy control group and in Post2 (**Figures 2G, H**). However, most of the points at Post2 overlapped with those in the healthy control group, suggesting similar bacterial community structures.

### Normalization of the gut microbiome in patients 12 months after SPD

To investigate the SPD-related changes in bacterial phylotypes in patients with cirrhosis, the microbial compositions of the stool samples from the groups were analyzed. The relative abundances (%) of the dominant microbial phyla, families, and genera clustered into each group are shown in **Figure S1**. At the phylum level, the gut microbiome composition of all individuals was mainly characterized by *Bacteroidetes*, *Firmicutes*, and *Proteobacteria*, with minor contributions from *Actinobacteria*, *Verrucomicrobia*, *Cyanobacteria*, *Fusobacteria*, and others. Although the bacterial community was highly diverse and there were marked interindividual differences, the microbial communities of the study patients differed from those of healthy controls. There were significantly lower relative abundances of *Bacteroidetes* and *Lentisphaerae* at Pre than in the healthy control group, while *Actinobacteria* was remarkably overrepresented (*P*<0.05) (**Figure S2**). However, the relative abundance of *Lentisphaerae* was recovered at Post2 (*P*<0.05) and was not significantly different from that in the healthy control group (*P*>0.05).

**Figure 3:**
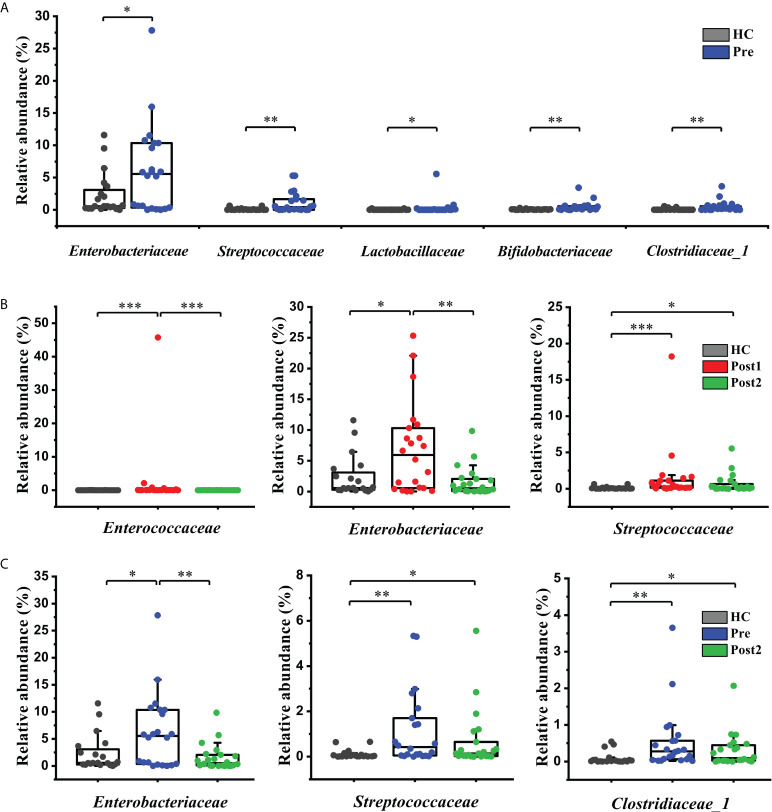
Microbiome phylotype alterations at the family level. **(A)** The relative abundances of five families were significantly different between the healthy control group (*n *= 20) and at Pre (*n *= 22). **(B)** *Enterococcaceae*, *Enterobacteriaceae*, and *Streptococcaceae* were significantly increased at Post1 (*n *= 22) compared with the healthy control group (*n *= 20) but showed a declining tendency at Post2 (*n *= 22). **(C)** *Enterobacteriaceae*, *Streptococcaceae*, and *Clostridiaceae_1* were reversed at Post2 (*n *= 22) compared with Pre (*n *= 22). The box plot illustration is provided in **Figure 2**, *P < 0.05, **P < 0.01, ***P < 0.001.

At the family level, the relative abundances of five families, including *Enterobacteriaceae*, *Streptococcaceae*, *Lactobacillaceae*, *Bifidobacteriaceae*, and *Clostridiaceae_1*, were significantly higher in the Pre group than in the healthy control group (*P*<0.05) (**Figure 3A**). Notably, three families containing many potentially pathogenic phylotypes, *Enterococcaceae*, *Enterobacteriaceae*, and *Streptococcaceae*, were significantly higher in abundance at Post1 than in the healthy control group (*P*<0.05) (**Figure 3B**). All three families showed a declining tendency at Post2 compared with Post1, especially as *Enterococcaceae* and *Enterobacteriaceae* were significantly reduced (*P*<0.01). *Enterobacteriaceae*, *Streptococcaceae*, and *Clostridiaceae_1*, the three disordered families at Pre, were reversed at Post2, and the relative abundance of *Enterobacteriaceae* even reached normal level when compared with the healthy control group (*P*>0.05) (**Figure 3C**). In addition, the correlation between disease severities and specific families at Pre revealed that the Child-Pugh score was negatively correlated with the relative abundance of the *Lachnospiraceae* (R=-0.502, *P*<0.05) and positively correlated with the *Streptococcaceae* (R=0.587, *P*<0.01) (**Figure S3A**). However, the relative abundances of the two families were reversed after the SPD at Post2, especially the difference in the *Lachnospiraceae* was statistically significant compared with the Pre (*P*<0.05) (**Figure S3B**). This was in accordance with the result that showed that the Child-Pugh score was dramatically reduced at Post2 (**Table 1**).

**Figure 4:**
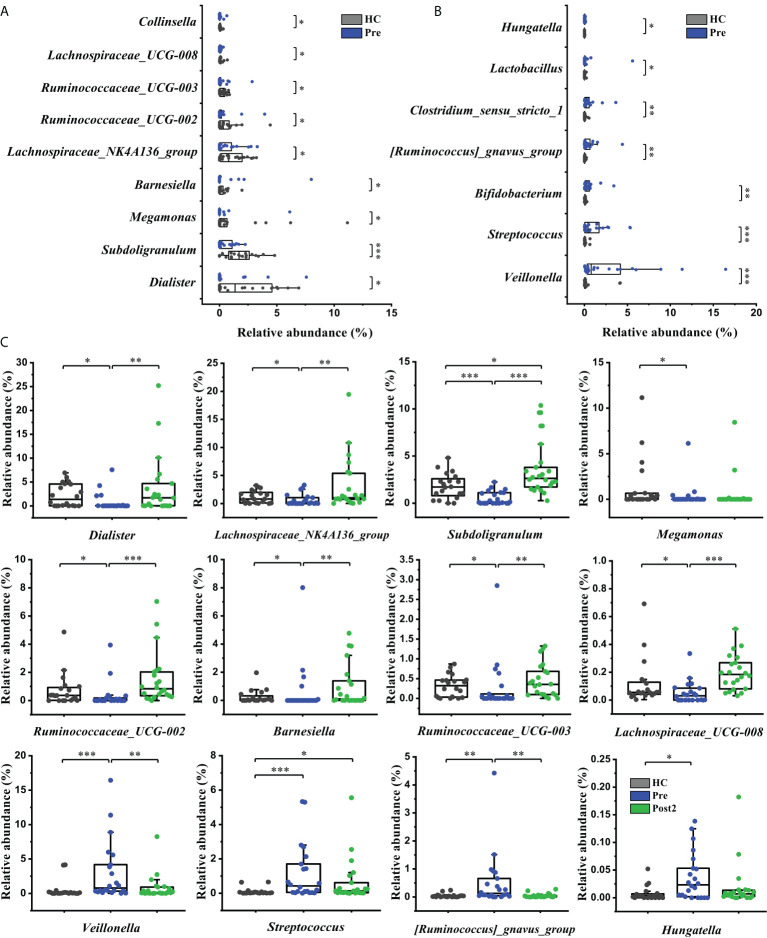
Microbiome phylotype alterations at the genus level. The phylotypes **(A)** decreased and **(B)** increased at Pre (*n* = 22) compared with the healthy control group (*n* = 20). **(C)** Twelve of the patient-associated genera had recovered at Post2 (*n* = 22). The box plot illustration is provided in **Figure 2**, *P < 0.05, **P < 0.01, ***P < 0.001.

The bacterial taxa were also compared at the genus level to further evaluate the differences between the groups. Sixteen cirrhosis-associated genera were differentially abundant between the Pre and the healthy control group (*P*<0.05). Among the nine genera enriched in the healthy control group (**Figure 4A**), *Dialister*, *Lachnospiraceae_NK4A136_group*, *Subdoligranulum*, *Ruminococcaceae_UCG-002*, *Barnesiella*, *Ruminococcaceae_UCG-003*, and *Lachnospiraceae_UCG-008* displayed significantly increased abundances at Post2 (*P*<0.01) (**Figure 4C**). In contrast, of the seven genera that were enriched at Pre (**Figure 4B**), *Veillonella* and *[Ruminococcus]_gnavus_group* were significantly decreased at Post2 (*P*<0.01) (**Figure 4C**). Altogether, the relative abundances of twelve cirrhosis-associated genera were improved at Post2, and, except for *Subdoligranulum* and *Streptococcus*, ten genera even reached normal levels (*P*>0.05).

**Figure 5:**
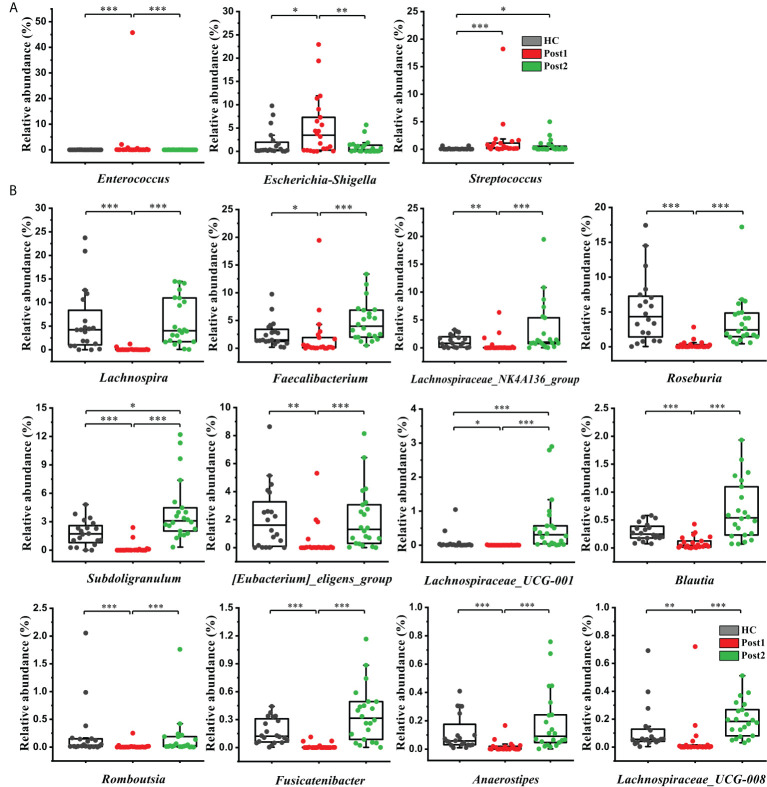
**(A)** Three opportunistic pathogens were enriched at Post1 (*n*=22) compared with the healthy control group (*n* = 20) but showed a declining tendency at Post2 (*n* = 22). **(B)** Twelve SCFA-producing genera were significantly suppressed at Post1 (*n* = 22), compared with the healthy control group (*n* = 20), and were restored after SPD at Post2 (*n* = 22). The box plot illustration is provided in **Figure 2**, *P < 0.05, **P < 0.01, ***P < 0.001.

In addition, it is worth mentioning that *Enterococcus*, *Escherichia-Shigella*, and *Streptococcus* were more enriched at Post1 compared with the healthy control group (**Figure 5A**). As concluded by many clinical studies, these genera contain many pathogenic species that are the leading causes of bacterial infections and are associated with a poor clinical prognosis in patients with cirrhosis (18, 21–23). The result of this study was in accordance with the changes in bacterial communities at the family level (**Figure 3B**). *Lachnospira*, *Faecalibacterium*, *Lachnospiraceae_NK4A136_group*, *Roseburia*, *Subdoligranulum*, *[Eubacterium]_eligens_group*, *Blautia*, and so on, which can produce beneficial substances called short-chain fatty acids (SCFAs) (24), were significantly suppressed in the Post1 group (**Figure 5B**). With the exception of *Streptococcus*, the relative abundances of these fifteen genera were reversed at Post2, and had significant differences compared with those at Post1 (*P*<0.01). All this evidence indicated an imbalance in the intestinal flora in cirrhotic patients. This situation was aggravated two weeks after the SPD. However, it had partly improved 12 months after surgery.

### The association of the restoration of the gut microbiome with the improvement of liver function and intestinal permeability after SPD

**Figure 6:**
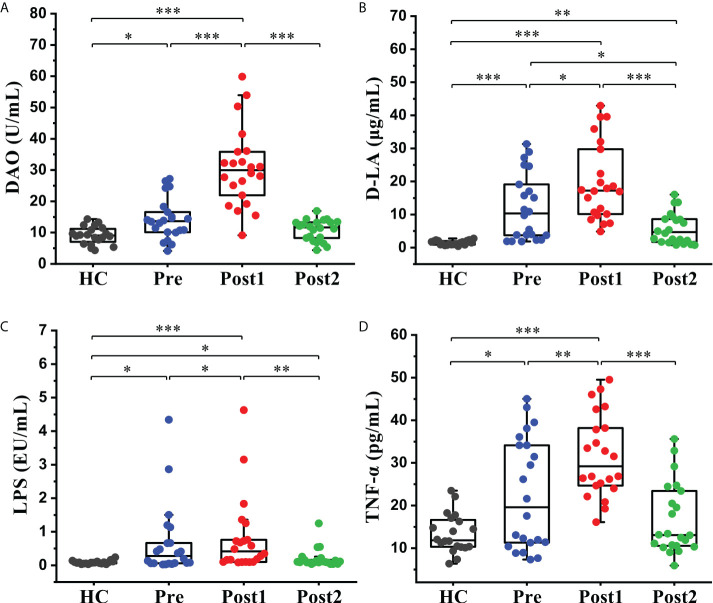
Alterations in the intestinal permeability and systemic inflammatory indices in peripheral blood. **(A)** Serum DAO, **(B)** D-LA, **(C)** LPS, and **(D)** TNF-α levels in the healthy control group (*n* = 20) and cirrhotic patients (*n*=22) before and after the SPD. Box plot illustration is provided in **Figure 2**, *P < 0.05, **P < 0.01, ***P < 0.001.

The concentration levels of DAO, D-LA, LPS, and TNF-α, which indirectly reflect intestinal permeability and systemic inflammatory levels, were measured to evaluate the effects of the SPD on intestinal permeability. As shown in **Figures 6A–D**, the levels of the four biomarkers were higher in Pre than in the healthy control group (*P*<0.05). Subsequently, these indices were further increased significantly at Post1 (*P*<0.05) and reached peak values. Finally, these indices displayed decreased levels at Post2 compared with Pre and Post1. The concentrations of D-LA and LPS in the Post2 group were still slightly higher than those in the healthy control group (*P*<0.05), but DAO and TNF-α were restored to a normal level (*P*>0.05).

**Figure 7:**
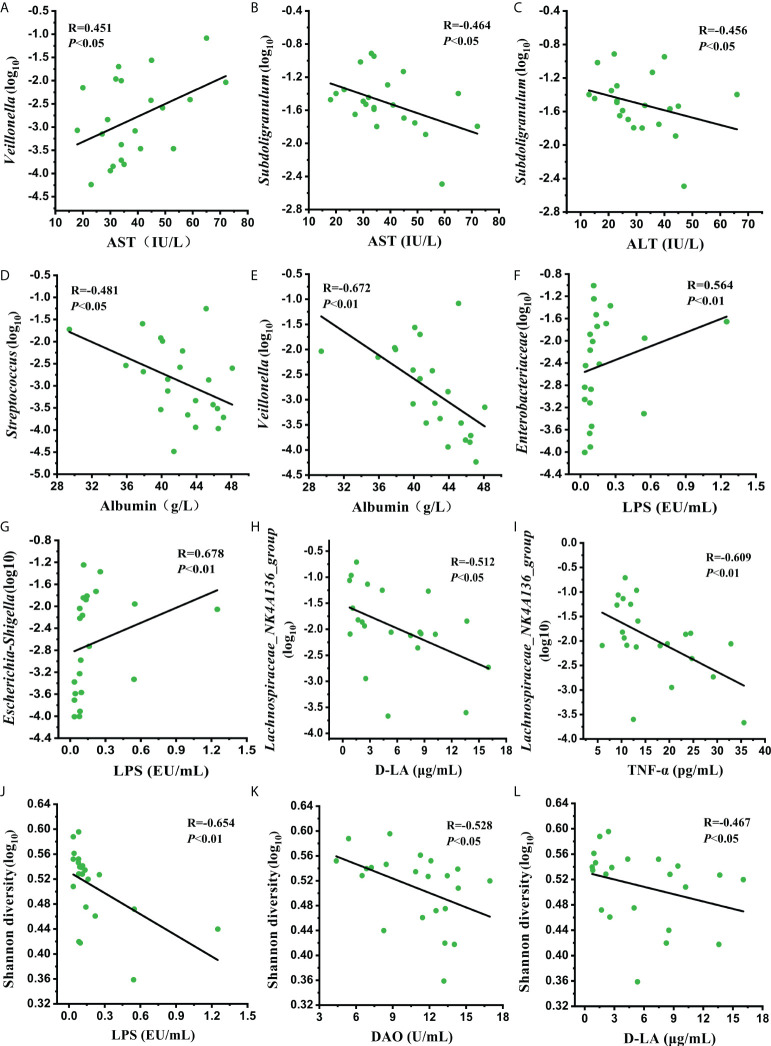
Correlation analysis of **(A–E)** the relative abundance of improved genera with liver damage and liver function indicators, **(F–L)** the relative abundance of improved bacterial taxa and Shannon diversity with intestinal permeability indicators at Post2.

To further elucidate whether the improved liver function and intestinal permeability were related to the recovery of the gut microbiome at Post2, a correlation analysis between the clinical parameters and improved genera was conducted. The results showed that the relative abundance of *Veillonella* was positively correlated with aspartate aminotransferase (AST) (R=0.451, *P*<0.05) (**Figure 7A**). The relative abundance of *Subdoligranulum* was negatively correlated with AST (R=-0.464, *P*<0.05) and alanine transaminase (ALT) (R=-0.456, *P*<0.05) (**Figures 7B, C**). The relative abundances of *Streptococcus* and *Veillonella* were negatively correlated with albumin (R=-0.481, *P*<0.01; R=-0.672, *P*<0.01, respectively) (**Figures 7D, E**). The relative abundances of *Enterobacteriaceae* and *Escherichia-Shihella*, which contain many kinds of gram-negative bacteria, were positively correlated with LPS concentration (R=0.564, *P*<0.01; R=0.678, *P*<0.01) (**Figures 7F, G**). The relative abundance of *Lachnospiraceae_NK4A136_group* was negatively correlated with D-LA (R=-0.512, *P*<0.05) and TNF-α (R=-0.609, *P*<0.01) concentrations (**Figures 7H, I**). In particular, the Shannon diversity of the gut microbiome showed a negative correlation with LPS (R=-0.654, *P*<0.01), DAO (R=-0.528, *P*<0.05), and D-LA (R=-0.467, *P*<0.05) concentrations (**Figures 7J–L**). Compared with the Pre group, the changing trends in these clinical parameters and bacterial taxa at Post2 were consistent with the correlations between them. These results suggested that normalizing the intestinal permeability through the restoration of some specific genera might ameliorate liver damage and its function.

## Discussion

To the best of our knowledge, this was the first prospective study to investigate the alterations in the gut microbiome and the intestinal permeability of HBV-related cirrhotic patients after undergoing an SPD. Our results revealed that gut microbial dysbiosis, increased intestinal permeability and impaired liver function were significantly mitigated at 12 months after surgery. Several improved clinical parameters were related to specific bacterial taxa with altered abundances.

Diversity is one of the essential tools by which to characterize the microbiome. Alpha diversity measures the diversity of the microbial community in a single sample, taking into account the number of different taxa and their relative abundances. A lower alpha diversity, which usually indicates a non-healthy and poor gut-microbial status, has also been reported to be associated with other diseases, such as inflammatory bowel disease, obesity, and colorectal cancer (25). Recent data suggest that mice with a complex gut microbiome showed reduced liver fibrosis in cholestasis-induced and toxin-induced liver injury, again demonstrating the health-promoting effects of a diverse gut microbiome (26). Beta diversity describes the degree of similarity in the microbial community composition between different samples. We found that the alpha diversity levels of the fecal microbiome exhibited a decreasing tendency in the HBV-related cirrhotic patients compared with healthy controls in this study. It was further reduced within two weeks of the SPD, probably due to conventional treatments during the perioperative period, such as antibiotic usage or abrosia. However, this situation improved 12 months after surgery. Meanwhile, the beta diversity of the fecal microbiome showed the same varying tendencies. Although the exact reason for the decreased microbial diversity in patients with cirrhosis is unclear, this phenomenon could be explained by the richness and evenness of microbial communities in the patients being insufficient to construct a rich and diverse biome such as those observed in healthy individuals. From the bacterial point of view, a more diverse community is associated with greater ecosystem resilience. The intestinal microenvironment may be more conducive to the overgrowth of certain bacteria that suppress other species below the detection threshold in patients under these abnormal conditions, thus decreasing bacterial diversity.

These alterations in diversity, which only indicated differences between the groups, did not define which taxa were responsible for such differences. Therefore, substantial differences in the gut microbiome that existed between the period before and after the SPD at different taxonomic levels were evaluated. At the phylum level, the relative abundance of *Bacteroidetes*, which is dominant in the human gut, was significantly reduced at Pre compared with the healthy control group. This was in accordance with previous studies that recruited cirrhotic patients with various etiologies (13, 14). The prognosis of inpatients with cirrhosis depends on the proinflammatory milieu, which may lead to organ failure. The proinflammatory milieu in cirrhosis is associated with the gut microbial dysbiosis characterized by an increase in the taxa belonging to the phylum *Proteoba*cteria (27). However, the relative abundance of *Proteoba*cteria was slightly higher in Pre than in the healthy control group, but the difference was not statistically significant. At a lower taxonomic level, *Lachnospiraceae*, which showed a negative correlation with the Child-Pugh score at Pre, was significantly overrepresented at Post2. In contrast, *Streptococcaceae*, which showed a positive correlation with the Child-Pugh score, exhibited the opposite result at Post2. These correlations are the key to discovering the connection between gut microenvironment variation and liver function improvement in cirrhotic patients.

Although illustrating the mechanism of underlying gut microbiome variations post-SPD was not the primary purpose of this study, we speculated that alleviating liver impairment in these patients could lead to this outcome. Splenic abnormalities are involved in the progression of liver fibrosis to cirrhosis through liver-spleen crosstalk. Splenic macrophages have been suggested as one of the crucial sources of transforming growth factor-beta1 (TGF-β1), which is considered the predominant fibrogenic cytokine in liver fibrosis (28). Splenic TGF-β1 plays a critical role in developing hepatic fibrogenesis. A splenectomy could decrease the serum level of TGF-β1 significantly while improving the parameters of liver fibrosis (29). Studies have also observed that a splenectomy promotes liver regeneration capacities of the liver by reducing TGF-β1 production and increasing hepatocyte growth factor levels (30, 31). In addition, the spleen influences the hepatic immune microenvironment by splenic soluble factor secretions and spleen-derived immune cell migrations. Our previous study demonstrated that splenic macrophages promoted chemokine CCL2 secretion in hepatic macrophages, facilitating monocyte recruitment and establishing an M1 dominant phenotype in hepatic macrophages, thus promoting hepatic fibrosis (32).

Liver function is impaired along with the reduction of bile acid secretion and increased intestinal pH, leading to intestinal bacterial overgrowth in patients with cirrhosis. One study showed that oral administration of bile acids could reduce bacterial overgrowth and prevent bacterial translocation and endotoxemia in cirrhotic rats, indicating that the status of liver function can directly impact the intestinal microenvironment (33). On the other hand, patients with liver cirrhosis may have elevated portal vein pressure, which causes intestinal mucosal congestion and edema, and reduced intestinal motility (9). Taken together, the dysfunction of the cirrhotic liver may change the intestinal microenvironment and cause gut microbiome imbalance. This study and others have shown that the SPD can reduce portal vein pressure and significantly improve liver function (4, 34), so as to better gut microenvironment, and thus further ameliorating the gut microbiome dysbiosis.

In the setting of cirrhosis, the intestinal barrier function is usually reduced due to impaired intestinal mucosal integrity and increased intestinal permeability. Elevated intestinal permeability is likely to cause translocation of pathogen-associated molecular patterns (e.g., LPS), which can induce a systemic inflammatory response and promote liver damage (11). One pattern, LPS, is a component of the gram-negative bacterial cell wall and plays a vital role in enterogenous infection. The gut microbiome is the primary source of the portal LPS, which can be recognized by the presence of toll-like receptor 4 in intestinal epithelial cells, promoting intestinal barrier injuries and liver fibrosis development (35). The intracellular enzyme, DAO, is confined primarily in intestinal villus cells that can catalyze the oxidation of diamines. Almost all of the DAO in the blood comes from the intestine (36). D-LA is a product of the bacterial fermentation of carbohydrates present in the intestinal lumen. Only a tiny amount of these substances can be detected in the serum under normal conditions, while concentrations rise rapidly when intestinal permeability is increased. Hence, the DAO, D-LA, and LPS concentrations have been considered sensitive biomarkers for reflecting intestinal permeability (37). Our study showed that intestinal dysbacteriosis was further aggravated at Post1 compared with Pre. On one hand, the dysbacteriosis increased the intestinal permeability so that *Bacteroidaceae* and *Enterobacteriaceae*, the gram-negative bacteria, which were significantly enriched at Post1, might rush into the circulation through the broken intestinal barrier and release their LPS in the bloodstream. The increased LPS levels subsequently cascaded the TNF-α to cause a stronger inflammatory response in the body. Additionally, our further correlation analysis revealed that the content of LPS was positively correlated with the content of TNF-α at Post1 (R=0.429, *P*<0.05) (data not shown). On the other hand, various gut-derived chemicals could more easily pass through the dysfunctional intestinal barrier and enter the systemic circulation from the intestines, leading to increased D-LA and DAO levels in peripheral blood. Notably, the diversity and complexity of intestinal microorganisms are critical to shaping intestinal barrier systems (38). A diverse gut microbiome is essential to regulating intestinal barrier function *via* the immune-mediated host defense response, which further prevents the progression of liver fibrosis (26). Our results showed that all these indicators at Post1 were obviously elevated compared with those at Pre but decreased compared with those at Post2. This was consistent with the changes in the gut microbiome diversity at different time points after the surgery. In the subsequent correlation analysis, we also confirmed that the Shannon diversity negatively correlated with DAO, D-LA, and LPS levels.

The gut microbiome plays an important regulatory role in maintaining the homeostasis of the intestinal mucosal barrier by resisting the colonization of pathogenic bacteria, promoting the secretion of intestinal mucin and sIgA, enhancing the tight junctions between intestinal epithelial cells, and regulating the differentiation of intestinal immune cells (39). The large intestine harbors commensal bacteria that ferment dietary fiber into short-chain fatty acids (SCFAs). SCFAs mainly consist of acetic acid, propionic acid, and butyric acid. They are an essential energy source for colonic enterocytes and goblet cells. Mucus secreted by goblet cells continuously replenishes the mucosal layer of the intestinal epithelium, serving as the first barrier against commensal bacteria and invading pathogens (40). The health-promoting functions of SCFAs for the host include inhibiting the inflammatory response, enhancing intestinal barrier function, and decreasing colonic pH and ammonia production. A decrease in SCFAs could result in hyperammonemia due to an increased pH and ammonia absorption in the gut (41), which is a very important pathogenetic factor in hepatic encephalopathy. We observed the interesting phenomenon that many SCFA-producing bacterial taxa, such as *Faecalibacterium*, *Roseburia*, and *Lachnospira*, were significantly decreased at Post1 compared with those at Post2. *Faecalibacterium prausnitzii* is a known gut bacterium that regulates mucus production by enhancing goblet cell differentiation and inducing gene expression in mucin glycosylation (42). It has also been found that butyrate, one of the primary metabolites of *Faecalibacterium prausnitzii*, can restore the number and function of mucin-secreting goblet cells by promoting the polarization of intestinal macrophages to M2 type, thereby promoting intestinal barrier repair (43). In addition, the flagellin of *Roseburia intestinalis* can recognize TLR5 and upregulate the tight junction protein Occludin and mucin MUC2 genes to recover intestinal barrier integrity (44).

With a decrease in SCFA-producing bacteria, *Escherichia-Shigella*, *Streptococcus*, and *Enterococcus*, the leading causes of opportunistic infections in patients with cirrhosis, were significantly increased at Post1. A healthy gut microbiome comprises diverse communities of commensal bacteria that mutually restrict and resist invading and colonizing pathogens (45). Consumption of these obligate anaerobes, resulting from, for instance, perioperative stress or antibiotics, can alter the utilization and downstream metabolism of microbiota-derived SCFAs by colonocytes. This change increases luminal oxygen availability, allowing the facultative anaerobes to expand (19). This may explain the prevalence of these genera in patients within two weeks after the SPD. A novel study has shown that *Escherichia coli* (belonging to the family *Enterobacteriaceae*) isolated from patients with liver cirrhosis can damage the intestinal barrier by reducing the expression of Occludin and E-cadherin (46). The present study found that the relative abundances of SCFA-producing bacteria at Post2 were significantly higher than those at Post1, while the relative abundance of *Enterobacteriaceae* was significantly lower, suggesting that the improvement of the gut microbiome 12 months after SPD may help to reduce intestinal permeability and improve intestinal barrier function. Improving intestinal microenvironment, in turn, may subsequently alleviate liver damage and improve liver function.

This study had some limitations. First, gut microbiome difference exists between cirrhotic patients with different etiologies (13). The present study focused on HBV-related cirrhotic patients, and the patients who did not meet the inclusion criteria for various reasons were excluded. Therefore, this study may not be sufficient to represent all cirrhotic patients with portal hypertension. It is necessary to perform additional sub-classification analysis based on different etiologies with more participants. Second, although the perioperative management was protocolized and consistent across most patients, the results could have been influenced by individual differences and unpredictable factors, which are common issues in this kind of study. Finally, the gut microbiome analysis was based on the 16s rRNA gene sequence, which can only identify the bacterial classification at the genus level, whereas metagenomic sequencing can reveal more accurate information at a species level and concerning microbial functions.

Even with these limitations, our work could still observe substantial differences in the gut microbiome and intestinal permeability between cirrhotic patients and healthy individuals. The differences were further exacerbated two weeks after the SPD. However, the patients then exhibited benefits that included the improvement of liver function and gut microenvironment 12 months after surgery. Improvements in the liver function and intestinal permeability were likely related to restoring the gut microbiome. Further studies are needed to determine whether and how the altered gut microbiome that occurs after an SPD influences the prognosis of patients.

## Data availability statement

The data presented in the study are deposited in the Sequence Read Archive (SRA) repository (https://www.ncbi.nlm.nih.gov/sra/), accession number: PRJNA838734.

## Ethics statement

The studies involving human participants were reviewed and approved by Institutional Ethics Committee of the Second Affiliated Hospital of Xi’an Jiaotong University. The patients/participants provided their written informed consent to participate in this study. Written informed consent was obtained from the individual(s) for the publication of any potentially identifiable images or data included in this article.

## Author contributions

YZ and ZL designed the research; XC and AZ collected the clinical data and samples; YZ, RZ, and YG performed the research; JW and BQ analyzed data; YZ and FJ wrote the first draft of the article; JG, GK, and ZL revised the article; all authors have read and approved the final version of the article.

## Funding

This work was supported by the Fundamental Research Funds for the Central University (No. XJJ2015102, XJJ2017190), the Free Exploration Project of Research Funds for the Second Affiliated Hospital of Xi’an Jiaotong University [2020YJ (ZYTS) 335], and the National Natural Science Foundation of China (No. 82100635).

## Acknowledgments

We thank Beijing Biomarker Technologies Corporation for their technical support in the sequencings. We would also like to thank Medjaden Inc. for the scientific editing of this manuscript.

## Conflict of interest

The authors declare that the research was conducted in the absence of any commercial or financial relationships that could be construed as a potential conflict of interest.

## Publisher’s note

All claims expressed in this article are solely those of the authors and do not necessarily represent those of their affiliated organizations, or those of the publisher, the editors and the reviewers. Any product that may be evaluated in this article, or claim that may be made by its manufacturer, is not guaranteed or endorsed by the publisher.
