# Supplementary material for: Improvement of gut microbiome and intestinal permeability following splenectomy plus pericardial devascularization in hepatitis B virus-related cirrhotic portal hypertension
Source: Front Immunol. 2022 Sep 8;13:941830. doi: 10.3389/fimmu.2022.941830 (PMC9493484; doi:10.3389/fimmu.2022.941830)
Supplement: Supplementary file 1 [file DataSheet_1.pdf]

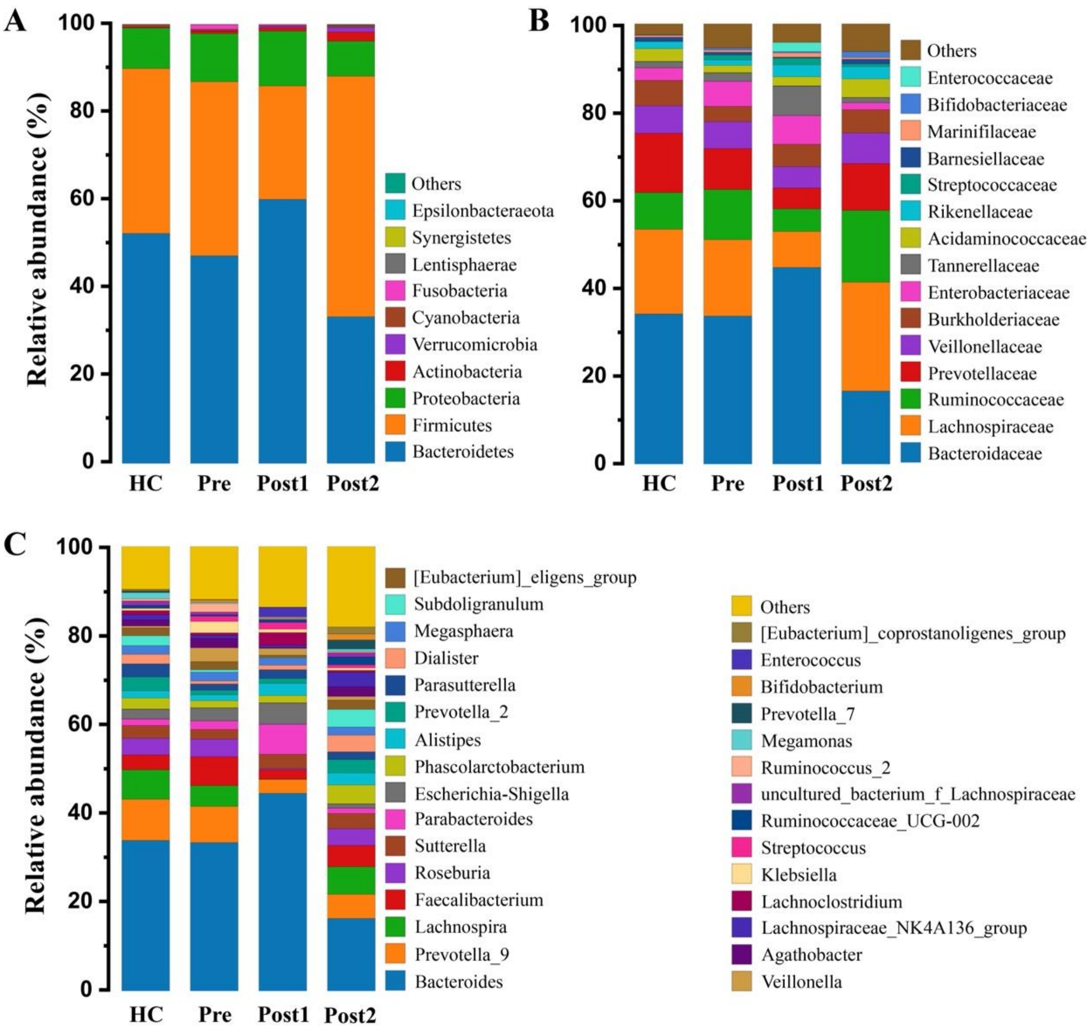

Figure S1. Taxonomic composition of the gut microbiome. The relative abundances of the gut microbiome at the **(A)** phylum, **(B)** family, and **(C)** genus levels in each group. All OTUs with relatively low abundances were grouped as “others”.

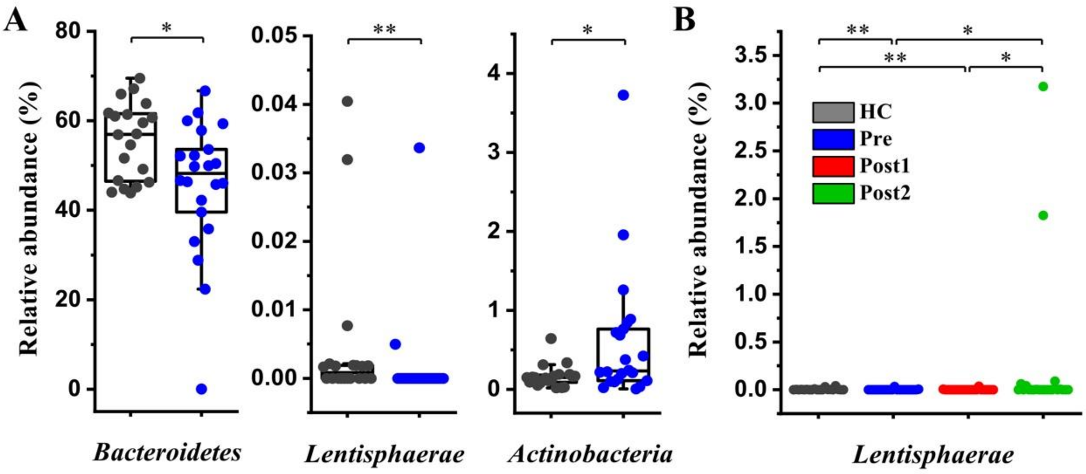

Figure S2. The microbiome phylotype alterations at the phylum level. **(A)** The relative abundances of *Bacteroidetes*, *Lentisphaerae*, and *Actinobacteria* were significantly different between the healthy control group (*n*=20) and Pre (*n*=22). **(B)** *Lentisphaerae* returned to normal levels at Post2 (*n*=22). Box plot illustration is provided in **Figure 2**, *P<0.05, **P<0.01.

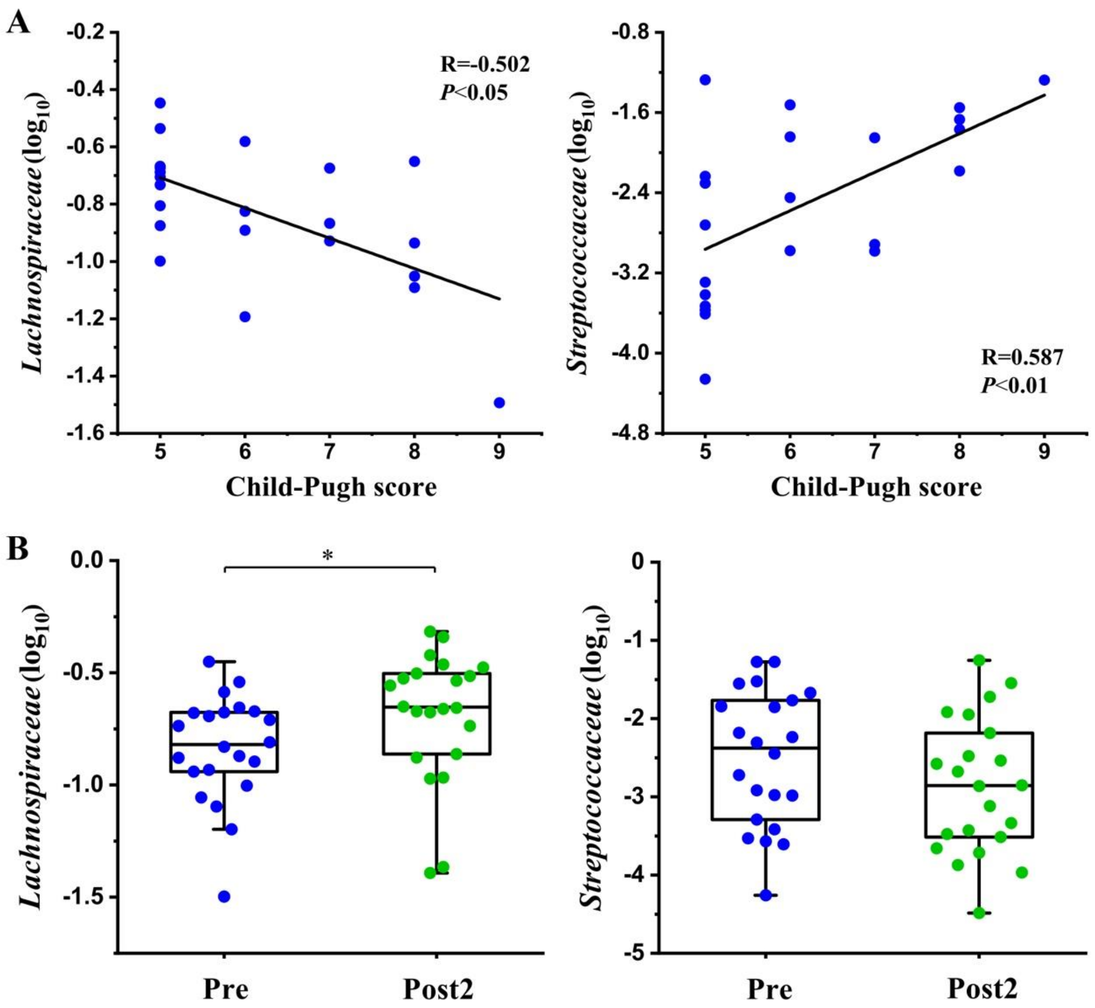

Figure S3. Correlations between Child-Pugh scores and bacterial abundances. **(A)** The Child-Pugh score was negatively correlated with the relative abundance of *Lachnospiraceae* and positively correlated with *Streptococcaceae* at Pre. **(B)** The abundances of the two families were reversed after the SPD at Post2 compared with Pre. The box plot illustration is provided in **Figure 2**, *P<0.05.
